# Intraocular Pressure Changes in Non-Glaucomatous Patients Receiving Intravitreal Anti-Vascular Endothelial Growth Factor Agents

**DOI:** 10.1371/journal.pone.0137833

**Published:** 2015-09-11

**Authors:** Weerawat Kiddee, Mayuree Montriwet

**Affiliations:** Department of Ophthalmology, Faculty of Medicine, Prince of Songkla University, Hatyai, Songkhla province, Thailand; Oregon Health & Science University, UNITED STATES

## Abstract

**Purpose:**

To study the prevalence of sustained intraocular pressure (IOP) elevation associated with intravitreal injection of anti-vascular endothelial growth factor (anti-VEGF) agents.

**Methods:**

Prospective comparative study. Non-glaucomatous patients scheduled to receive intravitreal injection of anti-VEGF therapy were recruited from an outpatient eye clinic, Songklanagarind Hospital between April 2013 and March 2014. The IOP was measured by Goldmann applanation tonometer before and at 1 hour, 1 week, 1 month, 3 months, and 6 months after injection. The IOP was compared using the repeated measures analysis. Sustained IOP elevation was defined as either an IOP > 21 mmHg or an increase from baseline ≥ 5 mmHg on two consecutive visits.

**Results:**

Seventy eyes of 54 patients met the inclusion criteria. The most common diagnosis was diabetic macular edema (48%). The mean IOP ± standard deviation (SD) before treatment was 13.7 ± 2.8 mmHg. The means ± SDs after treatment at 1 hour, 1 week, 1 month, 3 months, and 6 months were 11.3 ± 2.6, 13.7 ± 3.6, 14.1 ± 3.3, 14.0 ± 2.3, and 13.7 ± 2.4 mmHg, respectively. A mean of IOP difference at 1 hour postinjection and at baseline was −2.36 ± 2.5 mmHg (*P* < 0.001). Four of 70 treated eyes (5.7%) developed sustained IOP elevation (IOP ≥ 5 mmHg from baseline on two consecutive visits). The IOP returned to baseline levels after 1 month, in three eyes. One eye had sustained IOP elevation at 3 and 6 months follow-up. Thereafter, IOP returned to baseline level. There was no need of anti-glaucoma medication.

**Conclusions:**

After receiving intravitreal injection of anti-VEGF agent, a small proportion of non-glaucomatous eyes developed a sustained IOP elevation without requiring IOP-lowering treatment. At 1 hour postinjection, there was a significant reduction of the mean IOP compared with the baseline level.

## Introduction

Intravitreal injections of anti-vascular endothelial growth factor (VEGF) agents are commonly used to treat a variety of retinal and choroidal neovascular diseases. They have also emerged as the standard of care in the management of neovascular age-related macular degeneration (AMD) [1–3]. The well-established safety and efficacy of anti-VEGF intravitreal injection has resulted in its approval for the treatment of neovascular AMD and, more recently, retinal vein occlusion and diabetic retinopathy. The utilization of this treatment for these conditions has gained widespread acceptance worldwide [4, 5].

The introduction of additional fluid into the vitreous cavity by intravitreal therapy would be expected to cause an immediate rise in intraocular pressure (IOP). This transient, short-term IOP elevation (lasting up to 30 minutes) after intravitreal anti-VEGF therapy has been well described [6–9]. Although there is one study showing no significant changes in IOP [10], many studies showed effects of sustained anti-VEGF therapy on IOP elevation (occurring several weeks to months). The patients with increased IOP required anti-glaucoma drugs [11–21]. The present study therefore determined the prevalence of sustained IOP elevation associated with intravitreal injection of anti-VEGF agents in non-glaucomatous eyes.

## Materials and Methods

### Study design

A single-center, 6-month, prospective comparative study was carried out at the Department of Ophthalmology, Prince of Songkla University, from April 2013 to March 2014. The study was conducted in accordance with the tenets of the World Medical Association’s Declaration of Helsinki and was performed according to the Principles of Good Clinical Practice. The Institutional Review Board of Prince of Songkla University approved the study protocol. All patients provided written informed consent before participation in the study. Patient records/information was anonymized and de-identified prior to analysis. The trial was registered at the Clinical Trials Registry (NCT02474225).

### Subjects and data collection

The patients were scheduled to receive intravitreal injection of anti-VEGF agent [either bevacizumab (Avastin; Genentech Inc., San Francisco, CA, USA) or ranibizumab (Lucentis; Genentech Inc.)], and were recruited from an outpatient eye clinic, at Songklanagarind Hospital. Data included demographic information, ocular diagnosis, ocular surgeries, indications of injection, numbers of injection, intervals between injections, and IOP at each visit as measured using Goldmann applanation tonometry.

Inclusion criteria included individuals of 18–85 years of age, initial IOP < 21 mmHg, ability to understand and sign the consent form, and ability to follow the scheduled visit protocol. Exclusion criteria were open-angle or angle-closure glaucoma, suspected glaucoma (IOP > 21 mmHg and/or cup to disc ratio > 0.5), currently receiving a systemic beta blocker, previously receiving intravitreal injection of any medication (steroid, gancyclovir, and anti-VEGF agent), current use of steroid eye drops, and any ocular surface disease precluding a reliable IOP measurement.

### Surgical procedures

Intravitreal injections were performed in the operating room using aseptic techniques under topical anesthesia. Before injection, the eye was treated with antibiotic drops (topical 5% povidone—iodine solution). The intravitreal anti-VEGF injection was prepared by drawing up approximately 0.1 mL of bevacizumab (2.5 mg/0.1 mL) or ranibizumab (1 mg/0.1 mL) into a 1 mL tuberculin syringe. The excess was removed, and the remainder (1.25 mg of bevacizumab or 0.5 mg of ranibizumab/0.05 mL) was injected with a 30-guage needle through the superotemporal or superonasal pars plana at 3 mm or 4 mm posterior to the limbus, if the patient was pseudophakic or phakic, respectively. After injection, a sterile cotton swab was placed on the injection site to prevent reflux of fluid and vitreous. No paracentesis was performed before and after injection. After the procedure, patients were instructed to use antibiotic drops four times daily for 1 week.

### Follow-up evaluations and outcome measures

The IOP was measured before and at 1 hour after the injection. Follow-up visits were scheduled at postoperative 1 week and at months 1, 3, and 6. If the eye was scheduled to receive multiple injections, timing of IOP measurement was scheduled according to the first injection. In case of the eye receiving the 3-monthly injection protocol, the second injection was usually given at the study visit. At month 1 follow-up visit, the IOP measurement was obtained before the second injection of the protocol, to avoid the confounding effect from the short-term IOP rising. The same investigator (M.M.) obtained all IOP measurements. The mean IOP at each visit was obtained from the average of three measurements. The primary outcome measure was the proportion of eyes developing sustained IOP elevation defined as either an IOP > 21 mmHg or an increase from the baseline IOP of ≥ 5 mmHg on two consecutive visits. The criteria were chosen based on a previously published definition [10]. Secondary outcome measures included the means of IOP differences from baseline at each follow-up visit, incidence of intraoperative complications, and number of anti-glaucoma treatments required to treat the elevation of IOP.

### Statistical analysis

The analyses were performed with Stata software (version 12; StataCorp, College Station, TX, USA). Descriptive statistics were used to summarize patient demographics and baseline ocular characteristics. Repeated measures linear regression was used for IOP comparison. *P* values < 0.05 were considered statistically significant.

## Results

### Clinical characteristics

Seventy eyes of 54 patients were included in the present study. The mean age was 59 years (ranging from 30–80 years of age). The most common indication to receive anti-VEGF therapy was diabetic macular edema (48%). Demographic and baseline characteristics are summarized in Table 1. Most patients were treated with bevacizumab injection (91%); the remaining patients received ranibizumab injection. Sixteen (23%) of treated eyes received one injection. The rest of the studied eyes (77%) received more than one injection. Only 9 of 70 eyes (13%) received 4 or 5 injections. Most of treated eyes (64%) received multiple injections on a treat and extend protocol.

**Table 1:** Baseline demographic and ocular characteristic of study participants.

Age, year [mean, (range)]	59 (30–80)
Sex, n(%)	
Male	24 (44.4)
Female	30 (55.6)
Diagnosis, n(%)	
Exudative AMD	14 (25.9)
Diabetic macular edema	26 (48.1)
RVO with macular edema	11 (20.4)
IPCV	3 (5.6)
Treated eye, n (%)	
Right eye	20 (37)
Left eye	18 (33.4)
Both eyes	16 (29.6)
History of ocular surgery [total, n = 70 eyes, n (%)]	
No	61 (87.1)
Yes (cataract surgery, vitrectomy, pterygium excision)	9 (12.9)
Underlying systemic disease	
No	13 (24.1)
Yes (DM, HT, dyslipidemia, renal disease)	41 (75.9)

AMD = age-related macular degeneration; RVO = retinal vein occlusion; IPCV = idiopathic polypoidal choroidal vasculopathy; DM = diabetes mellitus; HT = hypertension; n = number (n = 54 patients).

### Outcomes

The mean ± standard deviation (SD) of IOP before treatment was 13.7 ± 2.8 mmHg. The mean IOPs ± SD after treatment at 1 hour, 1 week, 1 month, 3 months, and 6 months of follow-up were 11.3 ± 2.6, 13.7 ± 3.6, 14.1 ± 3.3, 14.0 ± 2.3, and 13.7 ± 2.4 mmHg, respectively. An IOP elevation of > 21 mmHg was observed in only 5.8% of study eyes at 1 hour postinjection. The maximal IOP rise was 23 mmHg. There was no sustained elevation of IOP using the criterion of an absolute IOP of > 21 mmHg on the following visit.

We observed that 8.7%, 5.8%, 5.1%, 1.9%, and 2.2% of the eyes had an IOP increase of ≥ 5 mmHg from the baseline IOP at 1 hour, 1 week, 1 month, 3 months, and 6 months follow-up postinjection, respectively. Only 4 of 70 eyes (5.7%) showed sustained IOP elevations (IOP ≥ 5 mmHg from baseline on two consecutive visits). All of these eyes were of different patients and were treated with intravitreal bevacizumab injection. Two eyes had IOP elevations at 1 week and 1 month postinjection. One eye showed IOP elevation at 1 hour and 1 week postinjection. Three eyes received three monthly injections (at baseline, month-1, and month-2); however they did not show sustained IOP elevation after 1 month post the first injection. Although the other eye received a single injection, it showed IOP elevations at 3 months and 6 months follow-up exams. Thereafter, IOPs returned to baseline levels. There was no need of anti-glaucoma medication.

The present study found a reduction in mean IOP at 1 hour, 1 month, and 3 months postinjections compared with the baseline IOP before the treatment (IOP ^post-injection–^IOP ^baseline^) (Table 2). However, the reduction of IOP after receiving intravitreal injection was statistically significant only at 1 hour postinjection [mean (SD), 2.36 (2.5) mmHg, *P* < 0.001]. In the present study, there was no report of complication, such as infection and bleeding, after the intravitreal injection of anti-VEGF agent.

**Table 2:** The mean of IOP difference from baseline IOP at each visit after receiving treatment.

Postinjection follow-up	IOP^postinjection^-IOP^baseline^ [Mean (SD), mmHg]	*P* value^a^
1 hour	-2.36 (2.5)	< 0.001
1 week	0.00 (3.1)	1.00
1 month	-0.37 (2.8)	0.59
3 months	-0.22 (2.7)	0.68
6 months	0.03 (2.6)	0.96

IOP = Intraocular pressure.

^a^Repeated measures linear regression.

## Discussion

The present prospective study of patients treated with intravitreal anti-VEGF agents shows a small proportion of eyes having sustained IOP elevation. Six of 70 eyes received ranibizumab intravitreal injection without a rise of IOP. The absence of increased IOP could be explained by the small sample size. However, 4 eyes (5.7%) showed sustained IOP elevation after treatment only with intravitreal bevacizumab. Initial IOP elevation varied from 1 hour, 1 week, and 3 months after treatment. Three eyes had IOPs that returned to baseline levels after 1 month, even when receiving monthly injections. Although the other eye received only a single injection, IOP elevation was shown at 3 and 6 month visits without an initiation of glaucoma treatment.

The possible mechanisms for sustained IOP elevation after intravitreal injection of anti-VEGF agents are not well understood [11]. Anti-VEGF agents may directly damage the trabecular meshwork [13]. However, a study of cultured human cells treated with bevacizumab did not demonstrate any toxic effects on trabecular meshwork cells [22]. Another possible mechanism is inflammation, such as drug-induced trabeculitis or uveitis [11]. Intraocular inflammation was not noted in any of the previous reported cases of delayed ocular hypertension (OHT), nor was it observed in the present study. It is possible that eyes in this series may have had some other unknown predisposition or risk for developing sustained IOP elevation after intravitreal treatment, but the current study did not show a direct causal relationship between intravitreal anti-VEGF therapy and sustained IOP elevation, due to the limited sample size and lack of controls.

Another possibility is that sustained IOP elevation may be a cumulative effect seen only after a large number of injections. Tseng et al. [18] have identified a high proportion of eyes that experienced large elevations in IOP only after many (more than 20 doses) cumulative intravitreal anti-VEGF injections. This is also a possible explanation for the low number of sustained IOP elevations observed in the present study. Other studies have reported sustained IOP elevations after an average of only four to five injections [12, 15], but this was not observed in our study. Since only 13% of the patients in our study received 4 or 5 injections and were followed for a short period of time after their initial injection, it is hard to compare to the previous observations. Good et al. [15] did not find an association between the development of sustained IOP elevation and the number of injections.

Kahook et al. [12] reported a patient with primary open-angle glaucoma in both eyes and AMD in the right eye. After a single bevacizumab intravitreal injection into the right eye, the IOP increased gradually over 5 months from 11 mmHg to 24 mmHg, while remaining in the low teens in the left eye. The underlying glaucoma may be a risk factor from increased IOP [12]. We did not observe these results because we excluded the glaucomatous patients. Wehrli et al. [10], however, reported that the incidence of delayed OHT after intravitreal anti-VEGF injection was low and did not differ between injected and control eyes, including eyes with glaucoma.

Another possible explanation for the low rate of sustained IOP elevation could be that 64% of patients in our study did not receive monthly injections, but rather, received an extended treatment regimen. It is possible that increasing the interval between injections may allow the anti-VEGF medication to be cleared from the eye. During injection, the anti-VEGF medication sometimes leaked from the injection site, and leakage of any vitreous contents, including anti-VEGF, could possibly eventually lead to decreased IOP shortly after the injection. However, we were unable to precisely record and quantify the amount of fluid leaking.

The strength of the present study is its prospective design. The limitations include the small sample size, the short duration of follow-up, the lack of control group, and no standardized of the injection technique.

Further studies with a greater sample size and longer follow-up periods should be considered to evaluate whether an increased number of anti-VEGF injections is associated with IOP changes.

In conclusion, our study showed that a small proportion of non-glaucomatous eyes developed sustained IOP elevations without requiring IOP-lowering treatment. There was a significant reduction in the mean IOP at 1 hour postinjection compared with IOP at baseline level.
